# Exome Sequencing Identifies a Novel FBN1 Variant in a Pakistani Family with Marfan Syndrome That Includes Left Ventricle Diastolic Dysfunction

**DOI:** 10.3390/genes12121915

**Published:** 2021-11-28

**Authors:** Nadia Farooqi, Louise A. Metherell, Isabelle Schrauwen, Anushree Acharya, Qayum Khan, Liz M. Nouel Saied, Yasir Ali, Hamed A. El-Serehy, Fazal Jalil, Suzanne M. Leal

**Affiliations:** 1 Department of Biotechnology, Faculty of Chemical and Life Sciences, Abdul Wali Khan University, Mardan 23200, Pakistan; farooqinadia2@gmail.com (N.F.); qayum.biotech007@gmail.com (Q.K.); yasirali@awkum.edu.pk (Y.A.); 2 Centre for Endocrinology, William Harvey Research Institute, Charterhouse Square Campus, Barts and The London School of Medicine and Dentistry, Queen Mary University of London, London EC1M 6BQ, UK; l.a.metherell@qmul.ac.uk; 3 Center for Statistical Genetics, Gertrude H. Sergievsky Center, Department of Neurology, Columbia University Medical Center, New York, NY 10032, USA; is2632@cumc.columbia.edu (I.S.); aa4471@cumc.columbia.edu (A.A.); liznouel1308@gmail.com (L.M.N.S.); 4 Department of Zoology, College of Science, King Saud University, Riyadh I1451, Saudi Arabia; helserehy@ksu.edu.sa; 5 Taub Institute for Alzheimer’s Disease and the Aging Brain, Columbia University Medical Center, New York, NY 10032, USA

**Keywords:** cardiovascular diseases, dilated cardiomyopathy, Marfan syndrome, left ventricular diastolic dysfunction, whole exome sequencing

## Abstract

Introduction: Cardiomyopathies are diseases of the heart muscle and are important causes of heart failure. Dilated cardiomyopathy (DCM) is a common form of cardiomyopathy that can be acquired, syndromic or non-syndromic. The current study was conducted to explore the genetic defects in a Pakistani family with cardiac disease and features of Marfan’s syndrome (MFS). Methods: A family with left ventricle (LV) diastolic dysfunction and MFS phenotype was assessed in Pakistan. The clinical information and blood samples from the patients were collected after physical, cardiovascular, and ophthalmologic examinations. An affected individual (proband) was subjected to whole-exome sequencing (WES). The findings were further validated through Sanger sequencing in the family. Results: Through WES and sanger validation, we identified a novel variant NM_000138.4; c.1402A>G in the Fibrillin-1 (*FBN1*) gene that segregates with LV diastolic dysfunction and MFS. Furthermore, bioinformatic evaluation suggested that the novel variant is deleterious and disease-causing. Conclusions: This study identified for the first time a novel *FBN1* variant in a family with LV diastolic dysfunction and MFS in Pakistan.

## 1. Introduction

Cardiomyopathies are a heterogeneous group of diseases that mostly result in progressive heart failure with significant morbidity and mortality. They represent an important and complex group of heart muscle disorders with heterogeneous phenotypic expression and multiple etiologies [1]. There are different causes of cardiomyopathies, most of them are genetic. These diseases are either part of a generalized systemic disorder or confined to the heart [2]. Dilated cardiomyopathy (DCM) is categorized into syndromic, non-syndromic and acquired types. Non-syndromic DCM is defined as DCM without other systemic involvement, while syndromic DCM is a multisystemic disorder [3], including those with mitochondrial myopathies, lysosomal storage disorders, and neuromuscular involvement [4]. Marfan syndrome (MFS) which involves the connective tissue and cardiovascular manifestations includes aortic dilation and ventricular arrhythmia [5].

Dilated cardiomyopathy is one of the major causes of heart failure and heart transplantation worldwide [6]. It is a myocardial disease involving dilation of the left ventricle, resulting in systolic dysfunction, heart failure, arrhythmia, and sudden cardiac death (SCD) [7]. DCM shows an autosomal-dominant inheritance pattern in ~90% of the cases and is X-linked or autosomal recessive in ~10% of cases, with variable penetrance and expressivity [8]. Furthermore, it is a genetically heterogeneous disease having the involvement of >100 genes [7,9]. More than 50 genes encoding the sarcomeric proteins, nuclear envelope, cytoskeleton, ion channels, sarcolemma and intercellular junctions have been associated with DCM [10]. Titin (*TTN)* is one of the important DCM genes, with truncating variants reported in about 25% of the familial form of DCM [11], while many other genes, such as *LMNA*, *MYH7*, *MYBPC3*, *TNNT2*, *MYH6*, and *SCN5A*, have been observed at lower frequencies [12].

MFS is a common autosomal-dominant inherited disorder of the fibrous connective tissues, involving prominent manifestations in many organ systems such as cardiovascular, skin, skeletal, ocular, pulmonary, and brain (dura mater abnormalities). MFS has a prevalence of 2 to 3 in 10,000 individuals [13,14]. Impaired left ventricular (LV) function in MFS occurs due to significant valvular heart disease [15,16]. The available data on left ventricular diastolic dysfunction in MFS are scarce [17,18]. MFS is mainly caused by heterozygous variants in the *FBN1* gene (OMIM #134797). *FBN1* encodes a 350 kDa glycoprotein member Fibrillin-1 of the Fibrillin family, which is a major constituent of 10–12 nm extracellular microfibrils that comprise the cardiac connective tissue scaffold and thus maintain the stability and assembly of the microfibrils [19,20]. The variants reported in the *FBN1* gene are responsible for 70–80% of MFS in general and ~90% of autosomal-dominant MFS cases [21,22]. More than 1800 *FBN1* variants have been associated with MFS, which are either predominantly inherited in an autosomal dominant fashion or are *de novo* in one-third of the patients [13].

To our knowledge, MFS with LV diastolic dysfunction has not been explored at the genetic level in the Pakistani population. Thus, we investigated if novel genes/variants can be involved in the genetic background of these cardiac diseases. Therefore, to test this hypothesis, we investigated a clinically well-characterized Pakistani family with LV diastolic dysfunction and skeletal features of MFS. A DNA sample from the proband of the family underwent WES and segregation of the identified variant was tested using Sanger sequencing. Furthermore, the identified variant was investigated for its effect on protein structure and function using *in silico* and bioinformatic tools. We identified a novel variant (NM_000138.4; c.1402A>G) in the *FBN1* gene with possible deleterious and damaging effects. This variant may indicate the etiologies of LV diastolic dysfunction and MFS in Pakistani patients.

## 2. Methodology

### 2.1. Family Recruitment and Clinical Diagnosis

**Figure 1:**
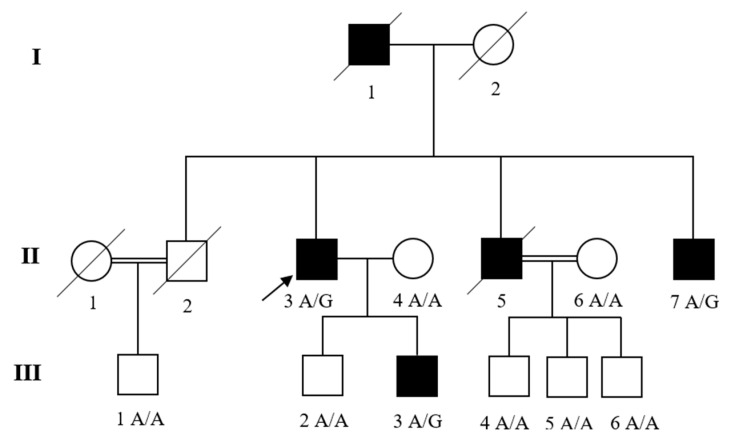
Pedigree of the family. Males and females are denoted by squares and circles, respectively. Filled symbols represent affected individuals and unfilled symbols unaffected family members. Crossed symbols indicate the family member is deceased. The arrow shows the proband. The proband (II-3) in the Family underwent exome sequencing while other members in this family underwent Sanger sequencing. The genotypes are shown for the *FBN1* variant [c.1402A>G: p.(T468A)].

A family (Figure 1) with LV diastolic dysfunction and MFS was recruited and selected for this study. The family was visited and interviewed for the clinical data and family history. Peripheral blood samples were collected from both affected and unaffected family members after obtaining written informed consent from every individual. The study was approved by the Institutional Review Boards (IRBs) of Abdul Wali Khan University Mardan, Mardan Medical Complex (MMC), Pakistan (IRB-AWKUM-2306) and Columbia University, USA (IRB-AAAS5864) in accordance with the declaration of Helsinki.

The affected family members were clinically evaluated and diagnosed by certified clinicians and cardiologists based on their family history, symptoms and medical records. The patients underwent detailed physical, cardiovascular, ophthalmologic, and skeletal system evaluations. They were examined by M-Mode and two-dimensional echocardiography, Doppler ultrasound and chest radiographs.

### 2.2. DNA Isolation and Whole Exome Sequencing

Genomic DNA was isolated via a GeneAll^®^ Exgene™ Blood SV mini-Kit (GeneAll Biotechnology Co., Ltd., Seoul, Korea) according to the manufacturer’s protocol. The DNA sample was fragmented using Covaris technology. The DNA fragments were amplified by ligation-mediated PCR (LM-PCR) and were hybridized to exome array for enrichment. After washing the non-hybridized fragments, the captured products were circularized. DNA Nanoballs (DNBs) were produced using the rolling circle amplification (RCA) technique. The resulting qualified captured library was loaded onto the BGISEQ platform.

The Agilent SureSelect Human All Exon V6 kit (Agilent Technologies, Inc., Santa Clara, CA, USA) was used for exome capture and preparation of exome libraries with a target region of 60.33Mb using DNA sample of the proband from the family. Exome sequencing was performed on the DNBseq™ platform, a BGISEQ-500 instrument (BGI Tech Solutions Hongkong Co., Ltd., Hong Kong, China) with 100 bp paired-end reads and mean sequencing depth of 74.81X on target regions.

### 2.3. Variant’s Annotation and Filtering

The WES reads were aligned to the human reference genome (GRCh38/hg38) by the Burrows–Wheeler Aligner software (BWAv0.7.15) [23,24,25]. PCR duplicate reads were removed with Picard tools. Local realignment around insertions/deletions (InDels), base quality score recalibration and variant calling were performed using the Genome Analysis Toolkit (GATK) [26,27].

ANNOVAR (v2019Oct24) was used for variant annotation [28]. Variants were filtered based on their frequency of occurrence in various databases (Genome Aggregation Database (gnomAD), Greater Middle East (GME), Kaviar Genomic Variant (kaviar_20150923), and Brazilian genomic variants (ABraOM)). Variants with a minor allele frequency (MAF) of <0.0005 were retained [29,30,31,32] and synonymous variants were excluded. Furthermore, various bioinformatics tools (CADD, SIFT, Polyphen-2, Mutation Taster, and Mutation Assessor included in dbNSFPv3.5 and dbscSNV1.1) were applied to predict the deleterious effect of the identified variant on protein structure and function (to predict the functional impact of the candidate variant). The conservation was examined using GERP++, PhyloP and PhastCons [33,34]. The databases including dbSNP, OMIM, ClinVar, HGMD, Genetics Home Reference (GHR) website, and Varsome were screened to determine whether the variant had any associated phenotypes.

### 2.4. Sanger Validation

The available DNA samples in the pedigree underwent Sanger sequencing to validate candidate variant obtained from WES and confirm its segregation with phenotype in the pedigree. Forward and reverse primers (FBN1-F-ACAGAATTACAACAGACCCTTGG; FBN1-R- GCCTTGCAAGCTCTGTAACC; Product Size: 521bp) were designed using Primer3 software [35]. The amplified products were purified by ExoSAP-IT (USB Corp., Cleveland, OH, USA) and were sequenced using the BigDye Terminator v3.1 Cycle Sequencing Kit followed by capillary electrophoresis on an ABI 3730 DNA Analyzer (Applied Biosystems Inc., Foster City, CA USA). The DNA sequences were then aligned to the reference genome sequence using the CodonCode Aligner v7.1.2 (CodonCode Corp., Centerville, MA, USA).

## 3. Results

### 3.1. Clinical Manifestations

The affected individuals of this family were observed and diagnosed with cardiac complications. They had common features of chest pain, shortness of breath, syncope, dizziness, and fatigue. It was a three-generation pedigree with three affected individuals II-3, II-7, and III-3 (Figure 1). The ages of onset were 58, 53, 42 and 16 years, respectively. The proband II-3 was a 63-year-old man, diagnosed with LV diastolic dysfunction, mitral valve regurgitation, tall thin stature, and long arms and legs. His parents were non-consanguineous and there was no family history of MFS. The proband’s father (I-1) died at the age of 80 years due to heart failure. Echocardiography (M-Mode/two-dimensional) of patients II-3, II-7, and III-3 confirmed LV diastolic dysfunction, normal-size cardiac chambers and normal LV ejection fraction. Mitral valve regurgitation or mitral insufficiency was also observed in patients II-3, and II-7. Patient II-5 died at the age of 57 years; however, his medical history was obtained from the clinical records. The physical examination of affected individuals II-3, II-7, and III-3 revealed skeletal features including tall thin stature, and long arms and legs. No other abnormalities were present in these patients. The clinical manifestations of the Family are given in Table 1.

### 3.2. Genetic Analysis

WES was carried out for the proband (II-3) of the family. The average sequencing depth in the target region was 74.81X. In addition, 99.62% of the target bases had 1X coverage and 98.05% of target bases had 10X coverage. The raw reads, clean reads, and mean sequencing depth (%) on the target region obtained from the selected individual were 89620140, 89445598, and 86.50, respectively. Furthermore, the variants were identified and filtered in the family.

### 3.3. Variant’s Validation and Segregation

**Figure 2:**
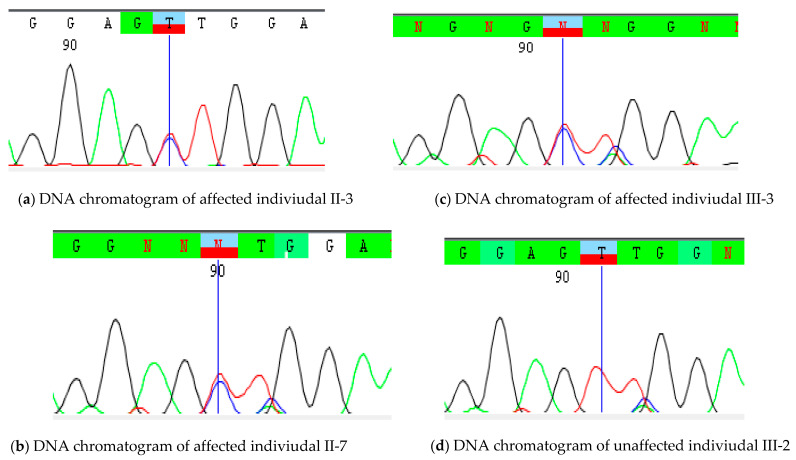
DNA sequence chromatograms of *FBN1* variant (NM_000138.4; c.1402A>G) obtained after Sanger sequencing. (**a**) DNA chromatogram of affected indiviudal II-3; (**b**) DNA chromatogram of affected indiviudal II-7; (**c**) DNA chromatogram of affected indiviudal III-3; (**d**) DNA chromatogram of unaffected indiviudal III-2.

The identified variant was subjected to Sanger validation. Thus, a novel variant [NM_000138.4; c.1402A>G: p.(T468A)] was identified in *FBN1* gene and was tested for segregation. Through Sanger sequencing, we found that family members II-7 and III-3 had the *FBN1* variant segregated with MFS in the Family and was classified as likely pathogenic according to American College of Medical Genetics (ACMG) guidelines. The DNA sequence chromatograms of the *FBN1* variant (NM_000138.4; c.1402A>G) are shown in Figure 2. 

### 3.4. Bioinformatic Analysis

**Figure 3:**
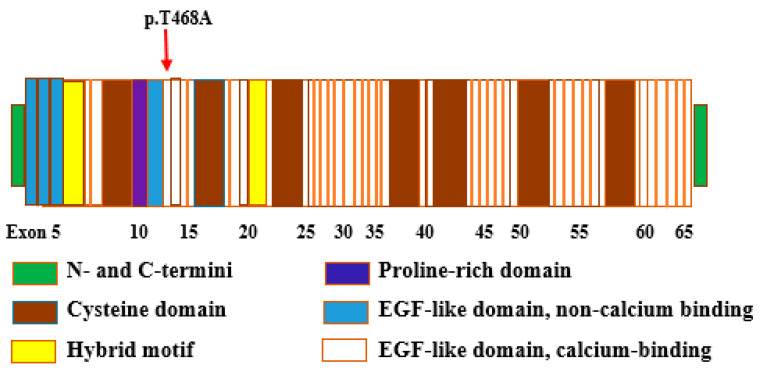
Schematic illustration of FBN1 (Fibrillin-1), a large glycoprotein (350 kDa, 2871 amino acids) with multiple functional domains. The figure shows that the variant p.(T468A) is located in cbEGF-like domain 3 of the protein [22].

The missense variant p.(T468A) in *FBN1* gene was designated to be damaging, deleterious and disease-causing by PROVEAN (Protein Variation Effect Analyzer), SIFT (Sorting Intolerant From Tolerant), FATHMM (Functional Analysis Through Hidden Markov Models), MutationTaster, and MutationAssessor. This variant is located in a highly conserved region of the calcium-binding epidermal growth factor-like domain 3 (cbEGF-like domain 3) of FBN1 protein and is predicted to affect protein function (Figure 3). 

## 4. Discussion

In this study, a family with LV diastolic dysfunction and MFS was evaluated, as shown in Figure 1. The affected individuals II-3, II-5, II-7 and III-3 displayed tall thin stature with long arms and legs, consistent with MFS, LV diastolic dysfunction, dyspnea, chest pain, fatigue, and syncope. Furthermore, no other abnormalities had been noticed in any of the affected family members. The proband from the family was subjected to WES analysis, and the variant observed was tested for segregation in other family members. Through WES data analysis we identified a novel variant (NM_000138.4; c.1402A>G) in the *FBN1* gene which segregated with MFS and LV diastolic dysfunction in the Family. The variant found in this study was analyzed using various online bioinformatic tools, such as PROVEAN, SIFT, FATHMM, MutationTaster, and MutationAssessor. Bioinformatic analysis suggested that the variant has deleterious and damaging effects. The *FBN1* variant replaces highly conserved threonine residue with alanine (NP_000129.3, p.T468A) in the calcium-binding epidermal growth factor (cbEGF)-like domain 3, and thus may affect the protein structure by disturbing the microfilament arrangement (Figure 3). The FBN1 protein comprises 47 epidermal growth factor-like (EGF-like) domains, 7 TGFβ-binding (TB) domains, 2 hybrid (Hyb) domains, N- and C-terminal domains. Each EGF-like domain consists of six highly conserved cysteines linked by three disulfide bonds (C1-C3, C2-C4, and C5-C6) [36]. The disruption of these bonds and the loss or addition of cysteine residue results in domain misfolding [37].

This variant was predicted to be deleterious by PROVEAN, SIFT, FATHMM, Mutation Taster, and MutationAssessor, with a CADD score of 20.2. Previously, many *FBN1* variants responsible for cardiovascular complications in MFS were identified [38]. In ClinVar, there are >2000 pathogenic or likely pathogenic *FBN1* variants. *FBN1* variants result in the loss of fibrillin-rich microfibrils in the cardiac extracellular matrix, and finally, weakness of the connective tissue. This results in the excessive activation of transforming growth factor-β (TGF-β), thus inducing phenotypic consequences in MFS [39,40,41]. Variants in exons 12–15 encoding cbEGF-like domains are responsible for a mild MFS phenotype (ectopia lentis) with late cardiovascular involvement [42]. However, a novel *FBN1* mutation previously found in a large Pakistani family with ocular and skeletal system defects had no cardiovascular features of MFS [43]. That said, a large number of variants in the *FBN1* gene have been associated with MFS, but the role of these variants in LV diastolic dysfunction has not been determined.

*FBN1* is expressed in many human tissues, including cartilage, tendon, cornea, zonules, and the cardiovascular system [44]. According to the Kyoto Encyclopedia of Genes and Genomes (KEGG) pathway annotation, the FBN1 protein is involved in the TGF-β signaling pathway (hsa04350). Besides their structural role, fibrillin microfibrils also contribute to tissue homeostasis through their interaction with cell surface receptors integrins and through interaction with growth factors, such as TGF-β and bone morphogenetic proteins (BMPs) [45,46]. Increased amounts of active TGF-β due to reductions in fibrillin have been reported to be implicated in MFS pathogenesis in mice with fibrillin1 deficiencies. Lung emphysema, vascular complications and bone overgrowth are the most distinctive features of mice with reduced expressions of the *FBN1* gene, and in MFS patients with heterozygous *FBN1* variants [47].

In conclusion, we report a novel variant (NM_000138.4; c.1402A>G) in *FBN1* gene associated with LV diastolic dysfunction and MFS in a Pakistani family. Our findings support the role of this variant in the genetic pathology of these cardiac diseases in the Pakistani population. Further investigation is needed to confirm the contribution of defects in the *FBN1* gene to these disease phenotypes and to elucidate the effects of this gene variant on function of the gene.

## 5. Web Resources

ANNOVAR, http://annovar.openbioinformatics.org/ (17 January 2020).

Burrows–Wheeler Aligner, http://bio-bwa.sourceforge.net/ (30 November 2018).

Combined Annotation-Dependent Depletion (CADD), https://cadd.gs.washington.edu/ (17 January 2020).

dbSNP, https://www.ncbi.nlm.nih.gov/projects/SNP/ (17 January 2020).

ClinVar, https://www.ncbi.nlm.nih.gov/clinvar (17 January 2020).

Exome Aggregation Consortium (ExAC), http://exac.broadinstitute.org/ (17 January 2020).

gnomAD, http://gnomad.broadinstitute.org/ (17 January 2020).

Genome Analysis Toolkit (GATK), https://software.broadinstitute.org/gatk/ (30 November 2018).

Genomic Evolutionary Rate Profiling (GERP), http://mendel.stanford.edu/SidowLab/downloads/gerp/ (17 January 2020).

MutationTaster, http://www.mutationtaster.org/ (17 January 2020).

Online Mendelian Inheritance of Man (OMIM), https://www.omim.org/ (17 January 2020).

PhastCons and PhyloP, http://compgen.cshl.edu/phast/ (17 January 2020).

Picard Tools, https://broadinstitute.github.io/picard/ (30 November 2018).

## Figures and Tables

**Table 1:** The echocardiographic parameters of the Family.

Patients	II-3	II-5	II-7	III-3
Age (years)	63	57	47	16
Age at the time of onset	58	53	42	16
Gender	Male	Male	Male	Male
Echocardiographic parameters				
LVEDD (mm)	46	50	53	42
LVEF (%)	65	71	59	60
FS (%)	35	40	32	36
MV E (m/sec)	0.86 ± 1.6	0.74 ± 1.4	0.83 ± 0.2	0.72 ± 1.9
MV A (m/sec)	0.56 ± 0.13	0.51 ± 0.11	0.47 ± 0.18	0.49 ± 0.15
E/A ratio	1.6 ± 0.5	1.8 ± 0.5	1.5 ± 0.6	1.6 ± 0.3
DTE (msec)	199 ± 32	171.6 ± 41.5	174.3 ± 63.2	165 ± 37.1
IVRT (msec)	73 ± 13	68 ± 15	72.4 ± 10	70.2 ± 14.1
Mitral valve regurgitation	+1	+1	+1	−
Skeletal system				
Height (cm)	185	187	178	173.58
Arm Span (cm)	196.1	198.22	190.46	184
Arm span to height ratio	1.06	1.06	1.07	1.06

LVEDD, left ventricular end-diastolic diameter; LVEF, left ventricular ejection fraction; FS, fractional shortening; MV E and MV A, mitral inflow velocities; DTE, deceleration time of mitral E wave; IVRT, isovolumic relaxation time.

## Data Availability

The data produced in this research is presented in this manuscript and will be publicly available.
